# Universal Framework for Quantum Error-Correcting Codes

**DOI:** 10.3390/e23080937

**Published:** 2021-07-23

**Authors:** Zhuo Li, Lijuan Xing

**Affiliations:** The State Key Laboratory of Integrated Services Networks, Xidian University, Xi’an 710071, China; lizhuo@xidian.edu.cn

**Keywords:** universal framework, quantum error-correcting codes, group algebra

## Abstract

We present a universal framework for quantum error-correcting codes, i.e., a framework that applies to the most general quantum error-correcting codes. This framework is based on the group algebra, an algebraic notation associated with nice error bases of quantum systems. The nicest thing about this framework is that we can characterize the properties of quantum codes by the properties of the group algebra. We show how it characterizes the properties of quantum codes as well as generates some new results about quantum codes.

## 1. Introduction

Quantum error-correcting codes provide a key approach to a scalable quantum computer that is resilient against decoherence and operational noise. Quantum errors can be expressed in terms of unitary error bases [1,2,3,4,5,6,7,8,9]. A particularly useful class of unitary error bases, called nice error bases, was introduced by Knill in [10], which is the foundation of the theory of quantum error-correcting codes [11]. Almost all of the quantum codes constructed so far are stabilizer or additive codes [3]. These are not the most general quantum codes, and there exist nonadditive quantum codes that are strictly better than any additive code [12]. Up to now, all good codes known have fallen into the class of what have been called pure quantum codes [11]. However, there are still some interesting and important problems about impure quantum codes [13]. Nearly all known quantum codes are constructed over finite fields [11]. However, it has been recognised that quantum codes can be constructed over finite rings as well [14].

When a new physical problem occurs, it is always desirable to find an appropriate framework for it, such as quantum mechanics for quantum physics. Since the occurrence of quantum codes, almost all researches are carried out on the specific types of quantum codes, for example, mainly on stabilizer codes, pure codes and codes over finite fields. In this paper, we are mainly interested in a universal framework for quantum codes, i.e., a framework that applies to all codes, no matter whether they are pure or not, stabilizer codes or not, over finite field or not. Firstly, we recall the properties of nice error bases. Then, we give the definitions of the group algebra and characters associated with nice error basis. Finally, based on the group algebra, we establish a universal framework for quantum codes. Through the discussion we show that this framework can characterize the properties of quantum codes as well as generate some new results about quantum codes. It is a powerful tool for use in future works on quantum codes.

## 2. Preliminaries

Quantum information can be protected by encoding it into a quantum error-correcting code. An ${\left((n,K,d)\right)}_{m}$ quantum code is a K-dimensional subspace of the state space of n quantum systems with m levels that can detect all errors affecting less than d quantum systems, but cannot detect some errors affecting d quantum systems.

Let $H=C^{m}$ be an m-level quantum system and let G be an additive group of order $m^{2}$ with identity element 0. A nice error basis of H is a set $E=\{E_{g}|g\in G\}$ of unitary operators on H such that

(i) $E_{0}$ is the identity operator,
(ii) $\mathrm{tr}E_{g}=0$ for all nonzero g∈G,
(iii) $E_{g}E_{h}=\omega_{g,h}E_{g+h}$ for all g,h∈G,

where complex numbers $\omega_{g,h}$ have modulus 1. G is called the index group of E. Moreover, $E_{n}≜E^{⊗n}=\{E_{g}≜E_{g_{1}}⊗\cdots ⊗E_{g_{n}}|g=(g_{1},\ldots ,g_{n})\in G^{n}\}$ is a nice error basis of n quantum systems $H^{⊗n}$.

**Lemma** **1.**  *If the index group G is Abelian, we have*

$$\sum_{g\in G}\omega_{g,h}{\bar{\omega }}_{h,g}=0$$

*for any nonzero h∈G.*

**Proof.**  By (iii) in the definition of nice error bases, it follows that

$$E_{a}E_{b}E_{h}=(\omega_{b,h}{\bar{\omega }}_{h,b})E_{a}E_{h}E_{b}=(\omega_{a,h}{\bar{\omega }}_{h,a})(\omega_{b,h}{\bar{\omega }}_{h,b})E_{h}E_{a}E_{b}$$

and

$$E_{a}E_{b}E_{h}=\omega_{a,b}E_{a+b}E_{h}=\omega_{a,b}(\omega_{a+b,h}{\bar{\omega }}_{h,a+b})E_{h}E_{a+b}=(\omega_{a+b,h}{\bar{\omega }}_{h,a+b})E_{h}E_{a}E_{b}$$

which means

(1) $$(\omega_{a,h}{\bar{\omega }}_{h,a})(\omega_{b,h}{\bar{\omega }}_{h,b})=\omega_{a+b,h}{\bar{\omega }}_{h,a+b}$$

Now, let $G_{h}=\{\omega_{g,h}{\bar{\omega }}_{h,g}|g\in G\}$. Then, from (1) $G_{h}$ is a subgroup of a cyclic group since all $\omega_{g,h}$ generate a cyclic group [15]. Thus, $G_{h}$ itself is a nontrivial cyclic group for any nonzero h∈G. □

In the next section, we provide the concept of the group algebra based on the nice error basis with the Abelian index group. For simplicity, we assume throughout the paper that the index group G is Abelian. This assumption is reasonable since such nice error basis exists for any finite dimensional quantum system [10].

## 3. Group Algebra

We are going to describe the elements of $E_{n}$ by formal polynomials in $z_{1},\ldots ,z_{n}$. In general, $E_{g}=E_{g_{1}}⊗\cdots ⊗E_{g_{n}}$ is represented by $z_{1}^{g_{1}}z_{2}^{g_{2}}\cdots z_{n}^{g_{n}}$, which we abbreviate $z^{g}$. We create the convention that $z_{i}^{g_{i}}z_{i}^{h_{i}}=z_{i}^{g_{i}+h_{i}}$. This forms the set of all $z^{g}$ into a multiplicative group denoted by Z. Thus, $G^{n}$ and Z are isomorphic groups, with addition in $G^{n}$

$$g+h=(g_{1},\ldots ,g_{n})+(h_{1},\ldots ,h_{n})=(g_{1}+h_{1},\ldots ,g_{n}+h_{n})$$

corresponding to multiplication in Z

$$z^{g}z^{h}=z_{1}^{g_{1}}\cdots z_{n}^{g_{n}}\cdot z_{1}^{h_{1}}\cdots z_{n}^{h_{n}}=z_{1}^{g_{1}+h_{1}}\cdots z_{n}^{g_{n}+h_{n}}=z^{g+h}$$

**Definition** **1.**  *The group algebra CZ of Z over the complex numbers C consists of all formal sums*

$$\sum_{g\in G^{n}}a_{g}z^{g},\to a_{g}\in C,z^{g}\in Z$$

Addition and multiplication of elements of CZ are defined in the natural way by

$$\sum_{g\in G^{n}}a_{g}z^{g}+\sum_{g\in G^{n}}b_{g}z^{g}=\sum_{g\in G^{n}}(a_{g}+b_{g})z^{g}$$

$$r\sum_{g\in G^{n}}a_{g}z^{g}=\sum_{g\in G^{n}}ra_{g}z^{g}r\in C$$

and

$$\sum_{g\in G^{n}}a_{g}z^{g}\cdot \sum_{h\in G^{n}}b_{h}z^{h}=\sum_{g,h\in G^{n}}a_{g}b_{h}z^{g+h}$$

To each $h\in G^{n}$ we associate the mapping $\chi_{h}$ from Z to the complex numbers given by

$$\chi_{h}(z^{g})=\mathrm{tr}E_{h}^{†}E_{g}^{†}E_{h}E_{g}/m^{n}$$

$\chi_{h}$ is called a character of Z. $\chi_{h}$ is extended to act on CZ by linearity

$$\chi_{h}(\sum_{g\in G^{n}}a_{g}z^{g})=\sum_{g\in G^{n}}a_{g}\chi_{h}(z^{g})=\sum_{g\in G^{n}}a_{g}\mathrm{tr}E_{h}^{†}E_{g}^{†}E_{h}E_{g}/m^{n}$$

Note that

(2) $$\chi_{h}(z^{g})=\prod_{i=1}^{n}\omega_{h_{i},g_{i}}{\bar{\omega }}_{g_{i},h_{i}}$$

Let

$$C=\sum_{g\in G^{n}}c_{g}z^{g}$$

be an arbitrary element of the group algebra CZ, with the property that

$$M=\sum_{g\in G^{n}}c_{g}\neq 0$$

**Definition** **2.**  *The transform of C is the element $C^{\prime }$ of CZ given by*

$$C^{\prime }=\frac{1}{M}\sum_{h\in G^{n}}\chi_{h}(C)z^{h}$$

*where χ was defined above.*

Suppose

$$C^{\prime }=\sum_{h\in G^{n}}{c^{\prime }}_{h}z^{h}$$

Then,

(3) $${c^{\prime }}_{h}=\frac{1}{M}\chi_{h}(C)=\frac{1}{M}\sum_{g\in G^{n}}c_{g}\mathrm{tr}E_{h}^{†}E_{g}^{†}E_{h}E_{g}/m^{n}\;\;\;\;h\in G^{n}$$

And ${c^{\prime }}_{0}=1$. Now, we describe several weight enumerators of the group algebra CZ. Let the elements of G be denoted by $\alpha_{0}=0,\alpha_{1},\ldots ,\alpha_{m^{2}-1}$, in some fixed order.

The first weight enumerator to be considered specifies the group algebra completely by introducing enough variables. In general, the variables $z_{ij}$ means that the $i^{\mathrm{th}}$ place in the vector g is the $j^{\mathrm{th}}$ element $\alpha_{j}$ of G. The vector $g=(\alpha_{a_{1}},\alpha_{a_{2}},\ldots ,\alpha_{a_{n}})$ is described by the polynomial

$$f(g)=z_{1a_{1}}z_{2a_{2}}\cdots z_{na_{n}}$$

Thus, g is uniquely determined by f(g). This requires the use of $nm^{2}$ variables $z_{ij}$, $1\leq i\leq n,\;0\leq j\leq m^{2}-1$.

What we shall call the exact enumerator of C is then defined as

$$E_{C}=\sum_{g\in G^{n}}c_{g}f(g)$$

Then, the exact enumerator of $C^{\prime }$ is

$$E_{C^{\prime }}=\sum_{h\in G^{n}}{c^{\prime }}_{h}f(h)$$

**Theorem** **1.**  $$E_{C^{\prime }}(z_{10},\ldots ,z_{ir},\ldots ,z_{n(m^{2}-1)})=\frac{1}{M}E_{C}\left(\sum_{s=0}^{m^{2}-1}\omega_{\alpha_{s},\alpha_{0}}{\bar{\omega }}_{\alpha_{0},\alpha_{s}}z_{1s},\ldots ,\sum_{s=0}^{m^{2}-1}\omega_{\alpha_{s},\alpha_{r}}{\bar{\omega }}_{\alpha_{r},\alpha_{s}}z_{is},\ldots ,\sum_{s=0}^{m^{2}-1}\omega_{\alpha_{s},\alpha_{m^{2}-1}}{\bar{\omega }}_{\alpha_{m^{2}-1},\alpha_{s}}z_{ns}\right)$$

**Proof.**  From (2) and (3), the LHS is equal to

$$\sum_{h\in G^{n}}{c^{\prime }}_{h}f(h)=\frac{1}{M}\sum_{g\in G^{n}}c_{g}\sum_{h\in G^{n}}\chi_{h}(z^{g})f(h)=\frac{1}{M}\sum_{g\in G^{n}}c_{g}\sum_{s_{1}=0}^{m^{2}-1}\sum_{s_{2}=0}^{m^{2}-1}\cdots \sum_{s_{n}=0}^{m^{2}-1}\prod_{i=1}^{n}\omega_{\alpha_{s_{i}},g_{i}}{\bar{\omega }}_{g_{i},\alpha_{s_{i}}}z_{is_{i}}$$

$$=\frac{1}{M}\sum_{g\in G^{n}}c_{g}\prod_{i=1}^{n}\sum_{s=0}^{m^{2}-1}\omega_{\alpha_{s},g_{i}}{\bar{\omega }}_{g_{i},\alpha_{s}}z_{is}$$

which is equal to the RHS. □

The next weight enumerator to be considered classifies vectors g in $G^{n}$ according to the number of times each group element $\alpha_{i}$ appears in g.

**Definition** **3.**  *The composition of $g=(g_{1},\ldots ,g_{n})$, denoted by comp(g), is $\left(s_{0},s_{1},\ldots ,s_{m^{2}-1}\right)$ where $s_{i}=s_{i}(g)$ is the number of components $g_{j}$ equal to $\alpha_{i}$. Clearly*

$$\sum_{i=0}^{m^{2}-1}s_{i}=n$$

We call the set {A(t)} the complete weight distribution of C where A(t) is the sum of $c_{g}$ with $\mathrm{comp}(g)=t=(t_{0},\ldots ,t_{m^{2}-1})$. We also define the complete weight enumerator of C to be

$$W_{C}(z_{0},\ldots ,z_{m^{2}-1})=\sum_{t}A(t)z_{0}^{t_{0}}\cdots z_{m^{2}-1}^{t_{m^{2}-1}}=\sum_{g\in G^{n}}c_{g}z_{0}^{s_{0}}\cdots z_{m^{2}-1}^{s_{m^{2}-1}}$$

Then, the complete weight distribution of $C^{\prime }$ is $\left\{A^{\prime }(t)\right\}$, where $A^{\prime }(t)$ is the sum of ${c^{\prime }}_{h}$ with $\mathrm{comp}(h)=t=(t_{0},\ldots ,t_{m^{2}-1})$, and the complete weight enumerator of $C^{\prime }$ is

$$W_{C^{\prime }}(z_{0},\ldots ,z_{m^{2}-1})=\sum_{t}A^{\prime }(t)z_{0}^{t_{0}}\cdots z_{m^{2}-1}^{t_{m^{2}-1}}$$

**Theorem** **2.**  $$W_{C^{\prime }}(z_{0},\ldots ,z_{r},\ldots ,z_{m^{2}-1})=\frac{1}{M}W_{C}\left(\sum_{s=0}^{m^{2}-1}\omega_{\alpha_{s},\alpha_{0}}{\bar{\omega }}_{\alpha_{0},\alpha_{s}}z_{s},\ldots ,\sum_{s=0}^{m^{2}-1}\omega_{\alpha_{s},\alpha_{r}}{\bar{\omega }}_{\alpha_{r},\alpha_{s}}z_{s},\ldots ,\sum_{s=0}^{m^{2}-1}\omega_{\alpha_{s},\alpha_{m^{2}-1}}{\bar{\omega }}_{\alpha_{m^{2}-1},\alpha_{s}}z_{s}\right)$$

**Proof.**  Set $z_{ij}=z_{j}$ for $1\leq i\leq n,0\leq j\leq m^{2}-1$ in Theorem 1. □

By setting certain variables equal to each other in the complete weight enumerator, we obtain the Lee and Hamming weight enumerators, which give progressively less and less information about the group algebra, but become easier to handle.

**Definition** **4.**  *Suppose now that $m^{2}=2\delta +1$ is odd, and let the elements of G be labeled $\alpha_{0}=0,\alpha_{1},\ldots ,\alpha_{\delta },\alpha_{\delta +1},\ldots ,\alpha_{m^{2}-1}$, where $\alpha_{m^{2}-i}=-\alpha_{i}$ for 1≤i≤δ. The Lee composition of a vector $g\in G^{n}$, denoted by Lee(g), is $\left(l_{0},l_{1},\ldots ,l_{\delta }\right)$ where $l_{0}=s_{0}(g)$, $l_{i}=s_{i}(g)+s_{m^{2}-i}(g)$ for 1≤i≤δ.*

We call the set {L(t)} the Lee weight distribution of C where L(t) is the sum of $c_{g}$ with $\mathrm{Lee}(g)=t=(t_{0},\ldots ,t_{\delta })$. We also define the Lee weight enumerator of C to be

$$L_{C}(z_{0},\ldots ,z_{\delta })=\sum_{t}L(t)z_{0}^{t_{0}}z_{1}^{t_{1}}\cdots z_{\delta }^{t_{\delta }}=\sum_{g\in G^{n}}c_{g}z_{0}^{l_{0}}z_{1}^{l_{1}}\cdots z_{\delta }^{l_{\delta }}$$

Then the Lee weight distribution of $C^{\prime }$ is $\left\{L^{\prime }(t)\right\}$, where $L^{\prime }(t)$ is the sum of ${c^{\prime }}_{h}$ with $\mathrm{Lee}(h)=t=(t_{0},\ldots ,t_{\delta })$, and the Lee weight enumerator of $C^{\prime }$ is

$$L_{C^{\prime }}(z_{0},\ldots ,z_{\delta })=\sum_{t}L^{\prime }(t)z_{0}^{t_{0}}z_{1}^{t_{1}}\cdots z_{\delta }^{t_{\delta }}$$

**Theorem** **3.**  *The Lee enumerator for the transform $C^{\prime }$ is obtained from the Lee enumerator of C by replacing each $z_{i}$ by*

$$z_{0}+\sum_{s=1}^{\delta }(\omega_{\alpha_{s},\alpha_{i}}{\bar{\omega }}_{\alpha_{i},\alpha_{s}}+{\bar{\omega }}_{\alpha_{s},\alpha_{i}}\omega_{\alpha_{i},\alpha_{s}})z_{s}$$

*and dividing the result by M.*

**Proof.**  Set $z_{m^{2}-i}=z_{i}$ for 1≤i≤δ in Theorem 2. □

The Hamming weight, or simply the weight, of a vector $g=(g_{1},\ldots ,g_{n})\in G^{n}$ is the number of nonzero components $g_{i}$, and is denoted by wt(g).

We call the set $\left\{A_{i}\right\}$ the Hamming weight distribution of C where $A_{i}$ is the sum of $c_{g}$ with wt(g)=i. We also define the Hamming weight enumerator of C to be

$$W_{C}(x,y)=\sum_{i=0}^{n}A_{i}x^{n-i}y^{i}=\sum_{g\in G^{n}}c_{g}x^{n-\mathrm{wt}(g)}y^{\mathrm{wt}(g)}$$

Then, the Hamming weight distribution of $C^{\prime }$ is $\left\{{A^{\prime }}_{i}\right\}$, where ${A^{\prime }}_{i}$ is the sum of ${c^{\prime }}_{h}$ with wt(h)=i, and the Hamming weight enumerator of $C^{\prime }$ is

$$W_{C^{\prime }}(x,y)=\sum_{i=0}^{n}{A^{\prime }}_{i}x^{n-i}y^{i}$$

**Theorem** **4.**  $$W_{C^{\prime }}(x,y)=\frac{1}{M}W_{C}(x+(m^{2}-1)y,x-y)$$

**Proof.**  In Theorem 2, put $z_{0}=x$, $z_{1}=z_{2}=\cdots =z_{m^{2}-1}=y$, and use Lemma 1. □

## 4. Universal Framework for Quantum Codes

In this section, we establish the universal framework for quantum codes based on the group algebra defined in the last section.

Given an arbitrary quantum code $C={\left((n,K,d)\right)}_{m}$, let $P=\sum_{i=1}^{K}|v_{i}\rangle \langle v_{i}|$ be the orthogonal projection onto C where $\left\{v_{i}\right\}$ is an orthonormal basis of C, and let G be the index group of any nice error basis E of the quantum system with m levels. Then, we can formulize the quantum code C as an element $C=\sum_{g\in G^{n}}c_{g}z^{g}$ from the group algebra CZ where

(4) $$c_{g}=\frac{1}{K^{2}}(\mathrm{tr}E_{g}P^{†})(\mathrm{tr}E_{g}^{†}P)=\frac{1}{K^{2}}{\left|\sum_{i=1}^{K}\langle v_{i}|E_{g}|v_{i}\rangle \right|}^{2}$$

We call C the element associated with the quantum code C in the group algebra CZ.

From (3), the transform of C is given by $C^{\prime }=\sum_{h\in G^{n}}{c^{\prime }}_{h}z^{h}$ where

(5) $${c^{\prime }}_{h}=\frac{1}{M}\sum_{g\in G^{n}}\frac{1}{K^{2}}(\mathrm{tr}E_{g}P^{†})(\mathrm{tr}E_{g}^{†}P)\mathrm{tr}E_{h}^{†}E_{g}^{†}E_{h}E_{g}/m^{n}\;=\frac{m^{n}}{K^{2}M}\mathrm{tr}E_{h}^{†}P^{†}E_{h}P=\frac{m^{n}}{K^{2}M}\sum_{i=1}^{K}\sum_{j=1}^{K}{\left|\langle v_{i}|E_{h}|v_{j}\rangle \right|}^{2}$$

where $M=\sum_{g\in G^{n}}c_{g}$. Since ${c^{\prime }}_{0}=1$, from (5), we obtain $M=m^{n}/K$. Thus,

(6) $${c^{\prime }}_{h}=\frac{1}{K}\sum_{i=1}^{K}\sum_{j=1}^{K}{\left|\langle v_{i}|E_{h}|v_{j}\rangle \right|}^{2}$$

From (4) and (6), and using the Cauchy–Schwartz inequality, we deduce that $c_{g}\leq {c^{\prime }}_{g}$ for all $g\in G^{n}$. Furthermore, from the definition of the minimum distance d we obtain that if K>1 then $c_{g}={c^{\prime }}_{g}$ for all g satisfying wt(g)<d and there exist some g with wt(g)=d such that $c_{g}\neq {c^{\prime }}_{g}$; if K=1 then $c_{g}={c^{\prime }}_{g}$ for all $g\in G^{n}$ and the minimum nonzero weight of g such that $c_{g}\neq 0$ is d.

So far we have established the universal framework for quantum codes:

For arbitrary quantum code $C={\left((n,K,d)\right)}_{m}$ we can characterize it as the element $C=\sum_{g\in G^{n}}c_{g}z^{g}$ of the group algebra CZ, called the element associated with the quantum code C, and the transform $C^{\prime }=\sum_{h\in G^{n}}{c^{\prime }}_{h}z^{h}$ of C so that

(1) the dimension K of C equals $m^{n}/M$ where $M=\sum_{g\in G^{n}}c_{g}$,
(2) the minimum distance d of C equals the minimum weight of g such that $c_{g}\neq {c^{\prime }}_{g}$ if K>1; the minimum nonzero weight of g such that $c_{g}\neq 0$ if K=1.

## 5. Conclusions

The nicest thing about the framework is that we can characterize the properties of quantum codes by the properties of the group algebra. So the problems about unfamiliar quantum codes can be transformed into those about familiar classical group algebra.

For example, we can define the weight distributions of the quantum code C as the weight distributions of the element C associated with C in the group algebra CZ and define the dual weight distributions of C as the weight distributions of the transform $C^{\prime }$ of C. Then, for any quantum code, its weight distributions and dual weight distributions must satisfy the identities in Theorems 1, 2, 3 and 4. Note that the results about exact enumerators, complete enumerators and Lee enumerators of quantum codes are completely new. For Hamming weight enumerators, the binary version was first proved for quantum stabilizer codes by Calderbank et al. in [16], and later generalized by Rains in [17]. The nonbinary version for stabilizer codes was proved by Ketkar et al. in [18]. The result given here is a generalization to the most general quantum codes.

Again, the purity of quantum codes can also be characterized by the group algebra. For arbitrary quantum code $C={\left((n,K,d)\right)}_{m}$, let $C=\sum_{g\in G^{n}}c_{g}z^{g}$ be the element associated with C in the group algebra CZ. Then C is pure if, and only if, $c_{g}=0$ for 0<wt(g)<d.

Finally if C is a quantum stabilizer code, from the definition of stabilizer codes, the element associated with C in the group algebra CZ can be written as $C=\sum_{g}z^{g}$ where the summation is over all such g that the operator $E_{g}$ belongs to the stabilizer of C. In addition, the transform of C can be written as $C^{\prime }=\sum_{h}z^{h}$ where the summation is over all such h that the operator $E_{h}$ belongs to the normalizer of C. Both forms imply the relationship between quantum stabilizer codes and classical codes.

Next, we will give some examples to illustrate the above conclusions. For qubits, it is well known that Pauli operators {I,X,Y,Z} constitute a nice error basis where

$$I=\;\left(\begin{array}{cc} 1 & 0\\ 0 & 1 \end{array}\right),X=\;\left(\begin{array}{cc} 0 & 1\\ 1 & 0 \end{array}\right),Y=\;\left(\begin{array}{cc} 0 & -i\\ i & 0 \end{array}\right),Z=\;\left(\begin{array}{cc} 1 & 0\\ 0 & -1 \end{array}\right)$$

In our terminology, the underlying Abelian index group of this nice error basis is G={0,x,y,z} satisfying x+x=y+y=z+z=0 and x+y=z, and the nice error basis can be denoted as $\left\{E_{0},E_{x},E_{y},E_{z}\right\}$ where $E_{0}=I,E_{x}=X,E_{y}=Y,E_{z}=Z$.

**Example** **1.**  *A nine-qubit code with a basis *

$$|v_{1}\rangle =(|000\rangle +\;|111\rangle )(|000\rangle +\;|111\rangle )(|000\rangle +\;|111\rangle )\;|v_{2}\rangle =(|000\rangle -\;|111\rangle )(|000\rangle -\;|111\rangle )(|000\rangle -\;|111\rangle )$$

*For this code, from (4) and (6), one can verify that $c_{g}={c^{\prime }}_{g}$ for all g with weight <3, for example, $c_{zz0000000}={c^{\prime }}_{zz0000000}=1$, and $c_{g}\neq {c^{\prime }}_{g}$ for some g with weight =3, for example, $c_{z00z00z00}=0$ but ${c^{\prime }}_{z00z00z00}=1$. Thus, the minimum distance d=3. Moreover, since there exists nonzero g with weight <d and $c_{g}\neq 0$, the code is impure. In fact, this code is the well-known ${\left((9,2,3)\right)}_{2}$ stabilizer code [1].*

**Example** **2.**  *A seven-qubit code with a basis *

$$|v_{1}\rangle =\;|0000000\rangle +{\left(|1011100\rangle \right)}_{\mathrm{cyc}}\;|v_{2}\rangle =\;|1111111\rangle +{\left(|0100011\rangle \right)}_{\mathrm{cyc}}$$

*where the subscript “cyc” indicates that all cyclic shifts occur. For this code, from (4) and (6), one can verify that $c_{0000000}={c^{\prime }}_{0000000}=1$, $c_{g}={c^{\prime }}_{g}=0$ for all nonzero g with weight <3, and $c_{g}\neq {c^{\prime }}_{g}$ for some g with weight =3, for example, $c_{0x000xx}=0$ but ${c^{\prime }}_{0x000xx}=1$. Thus, the minimum distance d=3. Moreover, since $c_{g}=0$ for all nonzero g with weight <d, the code is pure. In fact, this code is the well-known ${\left((7,2,3)\right)}_{2}$ CSS code [19].*

**Example** **3.**  *A five-qubit code with a basis *

$$|00000\rangle -{\left(|00011\rangle \right)}_{\mathrm{cyc}}+{\left(|00101\rangle \right)}_{\mathrm{cyc}}-{\left(|01111\rangle \right)}_{\mathrm{cyc}}$$

*together with all five cyclic shifts of *

$$\begin{array}{l} \;\;\;\;|00001\rangle -\;|00010\rangle -\;|00100\rangle -\;|01000\rangle -\;|10000\rangle \\ +\;|10011\rangle +\;|00111\rangle -\;|01110\rangle -\;|11100\rangle +\;|11001\rangle \\ +\;|10110\rangle -\;|01101\rangle +\;|11010\rangle -\;|10101\rangle -\;|01011\rangle \\ -\;|11111\rangle \end{array}$$

*For this code, from (4) and (6), one can verify that $c_{0000000}={c^{\prime }}_{0000000}=1$, $c_{g}={c^{\prime }}_{g}=0$ for all nonzero g with weight =1, and $c_{g}\neq {c^{\prime }}_{g}$ for some g with weight =2, for example, $c_{zz000}=0$ but ${c^{\prime }}_{zz000}=\frac{1}{3}$. Thus, the minimum distance d=2. Moreover, since $c_{g}=0$ for all nonzero g with weight <d, the code is pure. In addition, since ${c^{\prime }}_{zz000}=\frac{1}{3}$, the code must be nonadditive by the above conclusion. In fact, this code is the first nonadditive code ${\left((5,6,2)\right)}_{2}$ [12]. *

To sum up, we have presented a universal framework for quantum codes and shown how it characterizes the properties of quantum codes as well as generates new results about quantum codes. We can assert that this framework is a very useful and potential tool in studying the problems about quantum error-correcting codes. In fact, the idea of this framework has been used to solve the problem about the classification of perfect quantum codes [20]. Recently, we realized that this tool might have potential application in finding out various bounds on the parameters of the most general quantum error-correcting codes. We plan to develop this application further, among others, in our future works.
